# What Is the Clinical Significance of FDG Unexpected Uptake in the Prostate in Patients Undergoing PET/CT for Other Malignancies?

**DOI:** 10.1155/2013/476786

**Published:** 2013-12-28

**Authors:** Priya Bhosale, Aparna Balachandran, Raghu Vikram, Chitra Viswanathan, Homer Macapinlac, Eric Rohren, Ramanujan Prativadi

**Affiliations:** ^1^Body Imaging Section, Department of Diagnostic Radiology, Division of Diagnostic Imaging, The University of Texas MD Anderson Cancer Center, Houston, TX 77030, USA; ^2^Department of Nuclear Medicine, Division of Diagnostic Imaging, The University of Texas MD Anderson Cancer Center, Houston, TX 77030, USA; ^3^University of Buffalo School of Medicine, New York 14260, USA

## Abstract

*Purpose*. To determine the clinical significance of unexpected, abnormal FDG uptake in the prostate in patients undergoing FDG-PET/CT for staging of other primary malignancies without a prior history of prostate carcinoma. *Methods*. Retrospective search of FDG-PET/CT studies to identify patients with unexpected, abnormal FDG uptake in the prostate gland, who underwent subsequent biopsy, was performed. 26 patients were identified. Images were reviewed to determine the pattern of uptake within the prostate (focal or diffuse) and maximum standardized uptake value (SUVmax). PSA and Gleason scores were recorded. *Results*. 15/26 (58%) patients were found to have prostate carcinoma. Gleason scores ranged from 6 to 9.9. There was no statistical difference in the pattern of uptake (focal versus diffuse) or the SUVmax. Serum PSA levels with cancer (range, 2–26.8 ng; mean, 10.2 ng) and those without cancer (range, 2–10.5 ng; mean, 2.2 ng) were statistically significant (*P* < 0.007, Wilcoxon rank sum test). *Conclusions*. Patients with abnormal uptake in the prostate have a 58% likelihood of occult prostate cancer. In the setting of elevated serum PSA levels, abnormal prostate uptake should therefore be viewed with suspicion and a urology consult should be obtained; however, it is irrelevant in patients with underlying aggressive malignancies.

## 1. Introduction

Prostate cancer causes more deaths than any other cancer in men aged 55 to 74 years in many industrialized countries [1], and it is diagnosed incidentally in up to 28% of patients. The median age at diagnosis of prostate cancer is 67 years [2]. Prostate cancer is often regarded as an indolent malignancy and a disease that evolves over many years with aging; the premise for detecting and treating prostate cancer is a subject of debate.

Currently serial elevation of serum prostate-specific antigen (PSA) levels and an abnormal digital rectal exam raise the suspicion for prostate cancer.

Fluorodeoxyglucose (F-18 FDG) positron emission tomography (PET/CT) is an imaging modality that is, increasingly, being used for assessing cancer stage, response to therapy, and surveillance after treatment in various malignancies. Incidental uptake of FDG is occasionally noted within the prostate [3]; whether this finding is associated with prostate cancer or not is not adequately established. The purpose of this study was to determine the association between “incidentally” noted FDG uptake in the prostate gland and occult prostate carcinoma.

## 2. Patients and Methods

Institutional review board approval was obtained for this retrospective study. A search on the patients' database was performed for male patients who underwent F-18 FDG-PET/CT (PET/CT) imaging at our institution between April 1, 2006, and June 30, 2009, for known carcinoma other than prostate cancer (Figure 5); 1440 patients were identified. Results were further refined by dividing the group into patients with FDG uptake and patients without, yielding 124 patients. Two physicians with experience in reporting PET/CT reviewed all 124 cases and excluded all patients in whom the FDG avidity was thought to be secondary to the scatter from the bladder or urethral activity (Figure 6). Abnormally elevated FDG avidity was defined as any activity, which was more than that seen in background soft tissues. Patient's clinical records were then searched for any remote or documented history of prostate cancer and these were excluded from the study. Thirty-nine of the 65 patients did not have a biopsy and these were excluded from the study. All patients who had a sextant biopsy at our institution were included. The biopsy results, demographic data, and serum PSA values of these patients were recorded and tabulated on an excel spreadsheet (Microsoft, Redmond, WA, USA).

### 2.1. PET/CT Protocol

All PET/CT scans were performed per standard clinical protocol. Following a 6 hour fast, patients were administered FDG intravenously (mean 15 mCi 555 MBq; range, 8–20 mCi 444–740 MBq). Depending on the particular indication, imaging was performed 60–90 minutes after injection, extending from the vertex or orbits to the pelvis or feet. Scans were performed on General Electric (GE Medical Systems, Milwaukee, WI, USA) integrated PET/CT scanners with either BGO or LYSO detectors or 8- to 64-slice CT scanners.

For attenuation correction and diagnosis, CT without intravenous contrast was performed. The axial CT images were reconstructed to match the slice thickness of the PET images. PET scans were then acquired for 3–5 minutes per bed position, and these images were reconstructed to incorporate ordered subset expectation maximization with standard vendor-provided reconstruction algorithms. Attenuation data obtained from the CT component of the examination were used to attenuate correct PET images. To correct emission data for scatter, random events, and dead-time losses, the manufacturer's software was used.

### 2.2. Image Analysis

**Figure 1:**
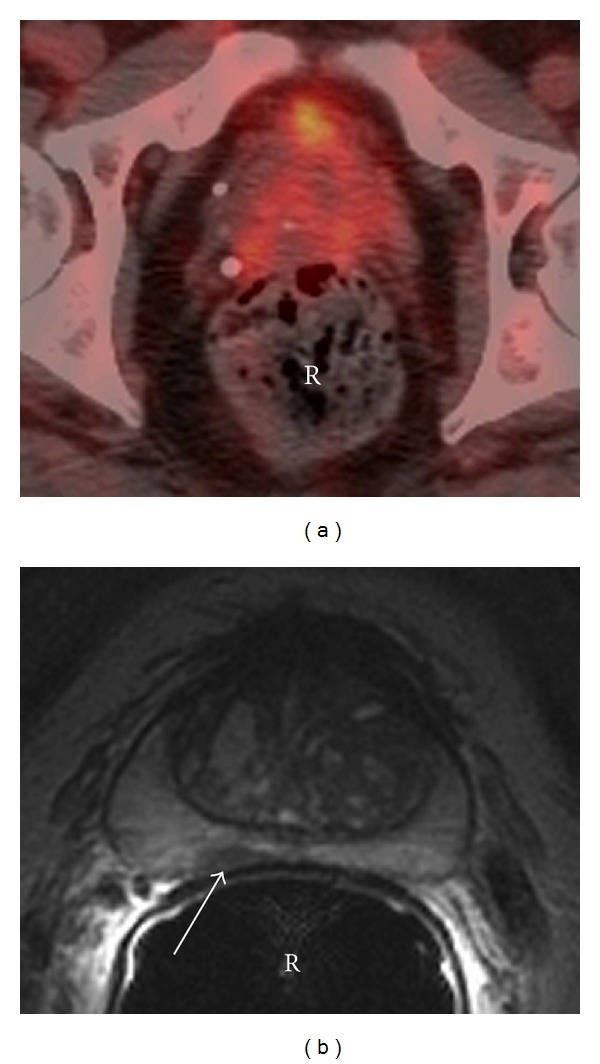
60-year-old male with history of Hodgkin lymphoma. (a) Axial fused PET/CT image shows diffuse uptake in the prostate. (b) Axial T2 weighted sequence through the prostate with an endorectal coil (EC) shows focal decreased signal in the peripheral zone posteriorly (arrow) anterior to the rectum (R). Biopsy of the prostate gland demonstrated prostate adenocarcinoma in the right and left base and the apex with a Gleason score ranging from 6 to 8.

**Figure 2:**
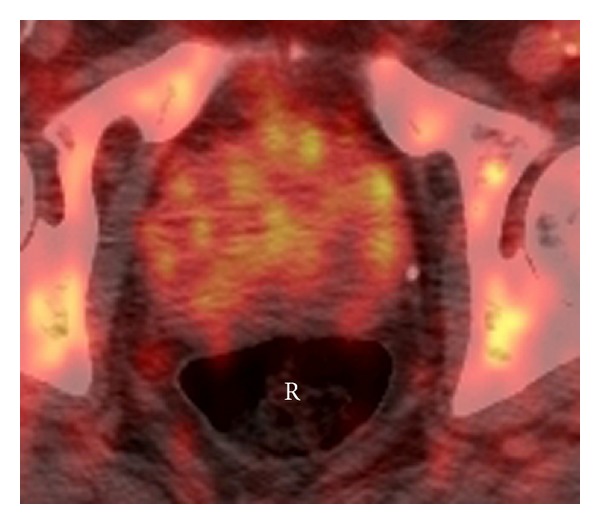
67-year-old male with a history lymphoma. Axial fused PET/CT image shows diffuse FDG uptake in the prostate anterior to the rectum (R). Subsequent biopsy was negative for cancer; however, the patient had BPH.

**Figure 3:**
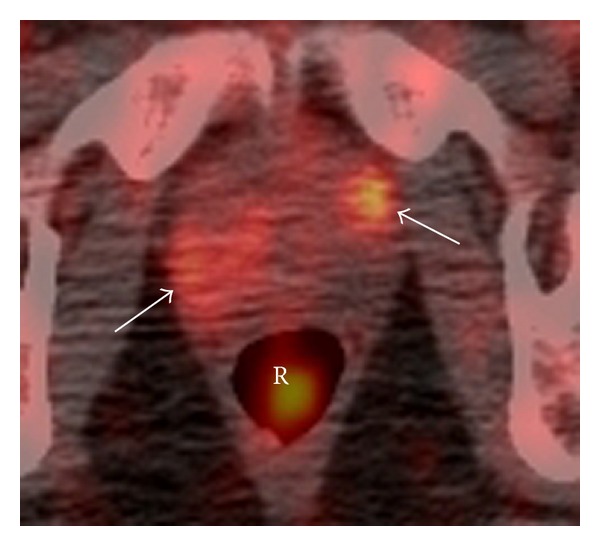
69-year-old male with history of an extra osseous osteosarcoma of the left calf. Fused axial PET/CT image shows two areas of focal uptake in the prostate gland (arrow) anterior to the rectum (R). Digital rectal exam was suspicious for a mass and the biopsy showed adenocarcinoma of the prostate with a Gleason score ranging from 8 to 9.

**Figure 4:**
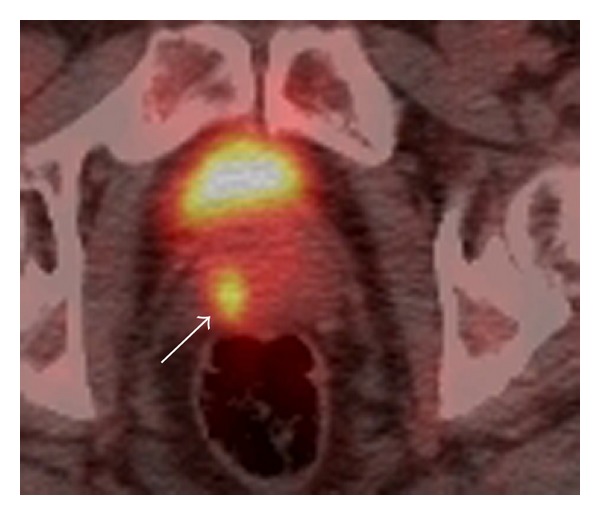
77-year-old male with a history of bladder cancer and follicular lymphoma. Axial fused PET/CT images show focal uptake in the prostate gland (arrow). Subsequent biopsy did not show evidence of malignancy but showed prostatitis.

**Figure 5:**
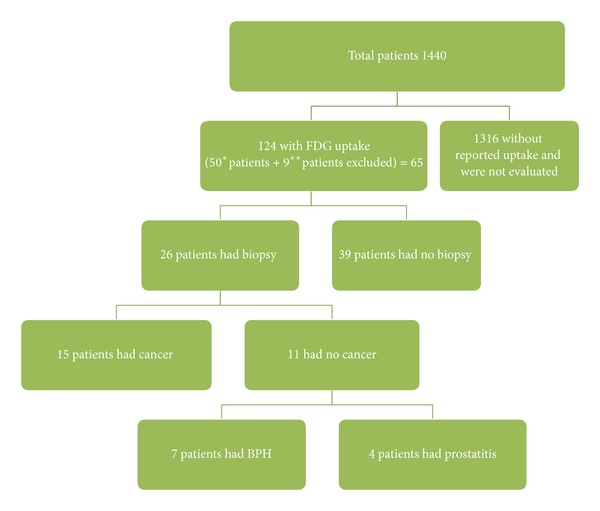
The number of patients evaluated and the results. *Patients were excluded as the uptake was not in the prostate but in the urethra and the scatter from the bladder. **Patients had history of prostate cancer.

Uptake in the prostate gland was classified as diffuse (Figures 1 and 2) or focal (Figures 3 and 4), and the maximum SUV measurements were recorded (SUVmax body weight calculation). Studies were reviewed on a workstation (Advantage Workstation, GE Healthcare), with reviewers blinded to histopathology results. Images were analyzed in multiple planes, and any discordancy in interpretation between the two readers was resolved by consensus. The results of the image review were correlated with the biopsy findings if present within 2 weeks of the PET study and with the PSA levels if present within 2 weeks of the PET scan.

### 2.3. Statistical Analysis

Summary statistics of PSA, SUV, Gleason score, and age in the form of mean, range, and SD were calculated for all patients and by group. Outcomes were compared between groups by the Wilcoxon rank sum test. Frequency table of FDG by patient group was tabulated, and Fisher's exact test was used to assess whether FDG outcome differed between the groups. Spearman's correlation test was performed to assess the correlation between SUVmax and Gleason score and between SUVmax and PSA. All tests were two-sided, and *P* ≤ 0.05 was considered statistically significant. Statistical analysis was carried out using SAS version 9 (SAS Institute, Cary, NC, USA).

## 3. Results

**Figure 6:**
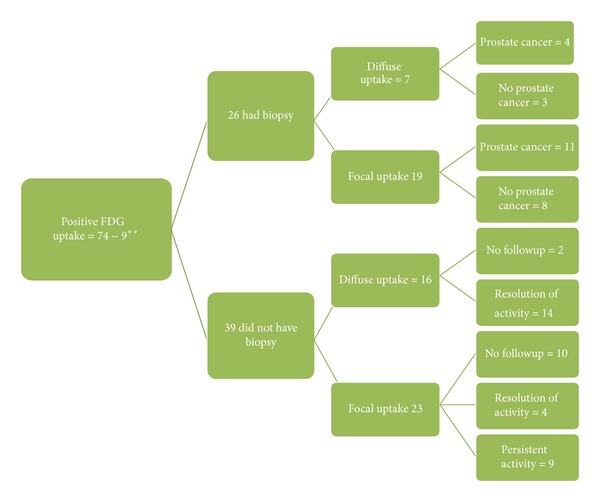
Patients with positive FDG activity in the prostate and their outcome. **Patients had history of prostate cancer.

Of the 39 patients with hypermetabolism in the prostate who did not have biopsy (Figure 6), 16 patients had diffuse uptake and 23 patients had focal uptake. The metabolic activity in 14 patients with diffuse uptake resolved on the subsequent PET/CT exam and 2 patients did not have followup. Of the 23 patients who had focal uptake, in 4 patients the activity resolved, 10 patients did not have a follow-up PET/CT, and in 9 patients it persisted; however no, further workup was done. Of these 9 patients with history of prostate cancer, had been treated with radiation and hormonal therapy and were not included in analysis and 5 patients had diffuse FDG uptake at the time of the PET/CT and 4 were not FDG avid.

Of the cohort, 26 patients underwent a transrectal biopsy. Fifteen of the 26 (58%) patients were diagnosed on subsequent transrectal biopsy with occult prostate carcinoma. In the other 11 patients, a diagnosis was made on either prostate biopsy, of benign prostatic hypertrophy (7 patients, 26%) or of prostatitis (4 patients, 15%). The median age of the patients was 74 years (range 53 to 90 years). Approximately 47% (7/15) of the patients with prostate cancer were between the ages of 65–74. There was no statistically significant difference between the means age of patients with and without prostate cancer after biopsy (*P* < 0.08). The median PSA for patients with cancer was 5.6 ng (range 1.8 to 26.8 ng), and the median PSA for patients without cancer was 1.70 ng (range 0.2 to 5.2 ng). Patients with prostate cancer had significantly higher PSA levels than patients without cancer (*P* < 0.007, Table 1); however, two patients with cancer had PSA < 4 ng.

There was no statistically significant difference between the SUVmax in patients with cancer and that in those without. In patients with prostate cancer there was no statistical difference between patients with focal and diffuse uptake (*P* > 0.99, Table 2); however, 11/19 (57.893%) patients with focal uptake did have cancer (Table 2). The Gleason score of the tumors ranged from 6.0 to 9.9 (mean 7.8, median 8.0). No correlation was found between the Gleason score and the SUVmax with that of range 2 to 19.70 (mean 8.0 and median 5.8) and no correlation was noted between PSA and SUVmax, with Spearman's rho equal to 0.55 (*P*—0.04) and −0.37 (*P*—0.12), respectively.

## 4. Discussion

Incidental FDG uptake in the prostate gland is an uncommon finding in PET/CT studies [4–8] and that was confirmed in the current study as incidental uptake it was reported in only 0.086% (124/1440) of PET/CTs. Nevertheless, in a busy practice, this finding is expected to be encountered with some frequency and often the dilemma exists as what one should do with such a finding. Newer agents such as ^11^C -Choline have shown promising results in identifying aggressive primary prostate cancers [9] and predict survival [10]. However, anti-3-(18)F-FACBC, a new synthetic amino acid compared to ^11^C –Choline, is superior in assessing disease recurrence in the setting of biochemical failure and perhaps will become the radiotracer of the future. These newer agents are not available for routine use and most centers rely on FDG for oncologic imaging.

It has been consistently reported that PET/CT has little role in the evaluation of the patient with newly diagnosed or recurrent prostate carcinoma confined to the prostate. This is primarily due to the low sensitivity of PET/CT for prostate carcinoma. In patients with elevated PSA, the sensitivity is reported to be in the range of 50 to 75% [11]. When incidental focal hypermetabolism is seen in the prostate gland, it is the specificity and the positive predictive value (PPV) of the imaging test that are at issue and not the sensitivity or the negative predictive value, and in our study we found that hypermetabolism in the prostate gland had a PPV of 58% for occult prostate carcinoma in patients.

Several parameters were assessed to determine the likelihood of malignancy in a particular patient with hypermetabolism in the prostate gland. Patient age had no correlation with malignancy nor did the intensity of FDG uptake (SUVmax). In addition, the literature suggests that uptake of FDG within the prostate may be due to poorly differentiated malignancy [12, 13] however, this was not seen in our study.

The only parameter, which had statistically significant association, was serum PSA level. An elevated serum PSA level in these patients further increased the likelihood of malignancy. An important finding in our study, however, is that two patients with prostate hypermetabolism and a PSA below the typical cutoff of 4 ng/mL used in large screening trials, ERSPC, and PLCO [14, 15] were found to have occult prostate cancer. Prostate biopsy was performed based on abnormal FDG activity in the prostate and an abnormal DRE. Therefore, although an elevated PSA in these patients increases the suspicion for prostate cancer, a “normal” PSA does not exclude the possibility of occult malignancy [16, 17].

We considered only 26/74 patients with indeterminate uptake, who had transrectal biopsy of the prostate and no known prostate cancer. In the absence of biopsy, the diagnosis in the remaining 39 patients is uncertain as a PSA serum was also not obtained. In 18 patients the uptake in the prostate gland resolved suggesting that this may have represented prostatitis, persisted in 9 patients, and may have been related to BPH or prostate cancer; however, it is doubtful that these longitudinal behaviors can be used to conclude with confidence the presence or absence of malignancy. The patients that were excluded from the study and did have persistent uptake did not have biopsy nor had their serum PSA evaluated due to presence of aggressive underlying malignancy such as metastatic melanoma and lung cancer. These patients may succumb to their underlying malignancy in a short period of time; uptake in the prostate has no clinical relevance as patients with low risk (Gleason lower than 6) can survive up to 16 years and with high risk prostate cancer (Gleason higher than 7) can survive up to 6 years [18]. However, patients with high Gleason score do require aggressive therapy [18].

Nearly three-quarters 11/15 (73%) of patients ultimately shown to have occult prostate cancer had focal nodular tracer uptake in the prostate gland, but focal versus diffuse uptake did not reliably differentiate malignancy from benign disease. Nonetheless, no matter what type of uptake is seen in patients who have nonaggressive underlying malignancies, this finding should be further evaluated, as incidentally found prostate cancers can also cause mortality in this once thought nonlethal disease [19, 20] and the treatment may be based on the grade, stage, and the age of the patient [18, 21]. Studies have shown survival benefit in patients who have incidental prostate cancers resected during cystoprostatectomy for bladder cancer [22] and such studies need to be performed in the setting of other malignancies. Specifically, future prospective PET/CT studies in the setting of underlying malignancies may help better define the significance of incidental focal prostate hypermetabolism. The results of this retrospective study support a larger, prospective trial, where in the setting of hypermetabolism seen in the prostate, a PSA should be obtained and if high, patients may benefit from a urology consult.

There are several limitations to this study. This is a retrospective study of patients who had PET/CT for underlying malignancy other than prostate cancer. In this study we only included patients who underwent biopsy of the prostate, and hence it was not designed to address the questions of sensitivity, specificity, or negative predictive value of PET/CT for the “detection” of occult prostate carcinoma. In addition, a control group was not present in this study to assess patients who did not have FDG uptake but had prostate cancer; however, this study was designed to answer only one question as to what should be done if FDG uptake is seen in the prostate.

In conclusion, the finding of incidental hypermetabolism in the prostate gland on PET/CT in oncologic patients is not definitely suggestive of malignancy. In patients with known malignancies, it depends on life expectancy, and the aggressiveness of the known malignancy as to whether the uptake within the prostate should be further evaluated. However, if the patient does have an indolent known cancer or is in remission, a serum PSA can be obtained, and if elevated, these patients may certainly benefit from a urology consultation.

## Figures and Tables

**Table 1:** The summary of outcomes of age, PSA, and SUV with prostate cancer using the Wilcoxon rank sum test.

	*N*	Mean	Std	Min	Median	Max	*P* value
PSA							
Prostate cancer							
No	9	2.20	1.71	0.20	1.70	5.20	0.007
Yes	11	10.23	8.75	1.80	5.60	26.80	
All	20	6.62	7.63	0.20	4.70	26.80	

SUV							
Prostate cancer							
No	10	8.41	4.41	2.00	7.25	15.70	0.58
Yes	14	8.01	6.02	2.00	5.85	19.70	
All	24	8.18	5.31	2.00	6.85	19.70	

Age							
Prostate cancer							
No	11	71.20	8.44	57.08	71.84	81.62	0.80
Yes	15	72.73	10.07	53.00	74.00	90.00	
All	26	72.08	9.27	53.00	73.50	90.00	

**Table 2:** The outcome of prostate cancer with respect to diffuse versus focal FDG activity using Fischer's exact test.

FDG	Cancer				*P* value
	No		Yes	
	*N*	%	*N*	%
Diffuse	3	42.86	4	57.14	>0.99
Focal	8	42.11	11	57.89	
